# Integrated thermal proteome and thermal proximity co-aggregation profiling identifies ATP6V1C1 as a novel anti-cancer drug target

**DOI:** 10.7150/ijbs.106843

**Published:** 2025-04-28

**Authors:** Shuang Zhang, Feng-Ming Li, Jun Wang, Yu Dong, Jing-Fang Luo, Xiao-Fang Huang, Yue Li, Guo-Yuan Zhu, Shi-Qian Qi, Han-Ming Shen, Qing Zhong, Chen Ming, Ke-Wu Zeng, Xiao-Jun Yao, Chris Soon Heng Tan, Jia-Hong Lu

**Affiliations:** 1 State Key Laboratory of Quality Research in Chinese Medicine, Institute of Chinese Medical Sciences, University of Macau, Macau SAR 999078, China.; 2 Department of Chemistry and Research Center for Chemical Biology and Omics Analysis, College of Science, Southern University of Science and Technology, Shenzhen, Guangdong 518055, China.; 3 Guangdong-Hong Kong-Macau Joint Lab on Chinese Medicine and Immune Disease Research, University of Macau, Macau SAR 999078, China.; 4 Department of Urology, Institute of Urology (Laboratory of Reconstructive Urology), State Key Laboratory of Biotherapy and Cancer Center, West China Hospital, Sichuan University, Chengdu, Sichuan, China.; 5 State Key Laboratory of Quality Research in Chinese Medicines, Guangdong-Hong Kong-Macao Joint Laboratory of Respiratory Infectious Disease, Macau Institute for Applied Research in Medicine and Health, Macau University of Science and Technology, Macau 999078, China.; 6 Faculty of Health Sciences, University of Macau, Macau 999078, China.; 7 Key Laboratory of Cell Differentiation and Apoptosis of Chinese Ministry of Education, Department of Pathophysiology, Shanghai Jiao Tong University, Shanghai 200025, China; 8 State Key Laboratory of Natural and Biomimetic Drugs, School of Pharmaceutical Sciences, Peking University, Beijing 100191, China.; 9 Dr. Neher's Biophysics Laboratory for Innovative Drug Discovery, State Key Laboratory of Quality Research in Chinese Medicine, Macau Institute for Applied Research in Medicine and Health, Macau University of Science and Technology, Taipa, Macau SAR 999078, China.; 10 Shenzhen Key Laboratory of Functional Proteomics, Guangming Advanced Research Institute, Southern University of Science and Technology, Shenzhen 518055, China.

**Keywords:** thermal proteome profiling, thermal proximity co-aggregation, V-ATPase, lysosome dysfunction, autophagy, tumor suppression

## Abstract

Bioactive natural products are invaluable sources for drug discovery. Unraveling their molecular targets uncovers the mechanisms of action and provides novel targets for drug development. However, the current approaches for target identification fall short in terms of efficiency, due to the extensive list of candidates and limited functional clues. Here we pioneer a strategy that integrates thermal proteome profiling and thermal proximity co-aggregation (TPP-TPCA) for high-efficient target identification. By linking functional targets to downstream perturbed protein complexes, this strategy enables a functional validation of candidate targets. For the first time, we applied this strategy to pinpoint the target of a natural compound veratramine (VAM) with anti-proliferation properties. Notably, the TPP identifies ATP6V1C1 as a candidate target of VAM, while TPCA reveals the dissociation of vacuolar (V)-ATPase. By directly binding to ATP6V1C1, VAM inhibits V-ATPase catalytic activity and lysosomal acidification, ultimately disrupting the autophagic-lysosomal pathway essential for cancer cell survival. Bioinformatics analysis reveals that ATP6V1C1 expression is upregulated in a variety of tumors and serves as a hub gene in breast cancer. Overall, this work presents an efficient strategy for target identification, demonstrating its successful application in identifying ATP6V1C1 as a promising target for cancer treatment.

## 1. Introduction

Natural products can offer powerful leads for therapeutic development, owing to their structurally optimized chemical scaffolds and ability to modulate the activities of various proteins. Nearly half (41.8%) of the new chemical entities approved by the FDA over the last 40 years were derived from or inspired by nature, especially in the area of cancer and infectious diseases 1-5. Deciphering the mechanism of action (MoA) for bioactive molecules identified from phenotypic screening and coincidental observation is crucial for further optimization in drug discovery 6. Natural products, in contrast to synthetic libraries, have a great deal of scaffold variation and structural complexity, which hinder MoA characterization. To address this challenge, various approaches, such as proteomic thermal shift assays and functional genomics approaches, have been employed 4, 7-9. However, finding the functional target of initial movement from a cluster of candidates remains a problem. To overcome this limitation, an unbiased, narrow-down approach (TPP-TPCA strategy) has been developed to identify the MoA of natural compounds 10, 11.

The TPP-TPCA strategy links putative targets identified with TPP (thermal proteome profiling) to downstream perturbed protein complexes revealed by TPCA (thermal proximity co-aggregation) 11, 12. TPP is a derivatization-free method that detects changes in thermal stability to identify ligand-binding proteins. It is widely used in drug target deconvolution and off-target research 11, 13. Biological activities are often carried out by assemblies of proteins termed protein complexes. Based on the coaggregation of proteins in complexes during heat denaturation, TPCA allows system-wide investigation of the temporal dynamics of protein complexes. Integrating TPP and TPCA could link binding targets (molecular initiating event) to downstream perturbed protein complexes and pathways before the onset of transcription response, pinpointing actual functional targets responsible for MoA, particularly for natural products.

Here, we applied TPP-TPCA strategy to investigate the MoA of VAM, an alkaloid sourced from the roots and rhizomes of *Veratrum.* For generations, the *Veratrum* species was used to cure jaundice, chronic malaria, and aphasia emerging 14-16. Recently VAM was reported to exert protective effects including antihypertensive, anti-proliferative activity 17-20, and we verified this function in breast cancer cell lines. However, the anti-proliferation mechanism remains unclear. Our integrated profiling revealed a subset of perturbed protein complexes involved in degradation via vesicle-mediated transport pathway, oxidative metabolism process, and biosynthesis of protein.

Notably, we identified a dissociated complex regulated by VAM, vacuolar H^+^-ATPase (V-ATPase), which are proton pumps responsible for acidifying lysosomes to maintain the acidic environment and facilitating autophagosome-lysosome fusion 21-24. Also, we uncovered ATP6V1C1, a catalytic subunit of V-ATPase, as a functional target of VAM and verified the binding of VAM to ATP6V1C1 result in the inactivation of V-ATPase and dysfunction of the autophagic-lysosomal system. These results highlight ATP6V1C1 as a functional target and underscore the TPP-TPCA strategy's potential for elucidating natural compounds' MoA.

## 2. Results

### 2.1 Integrated TPP-TPCA strategy to decipher MoA of VAM

**Figure 1:**
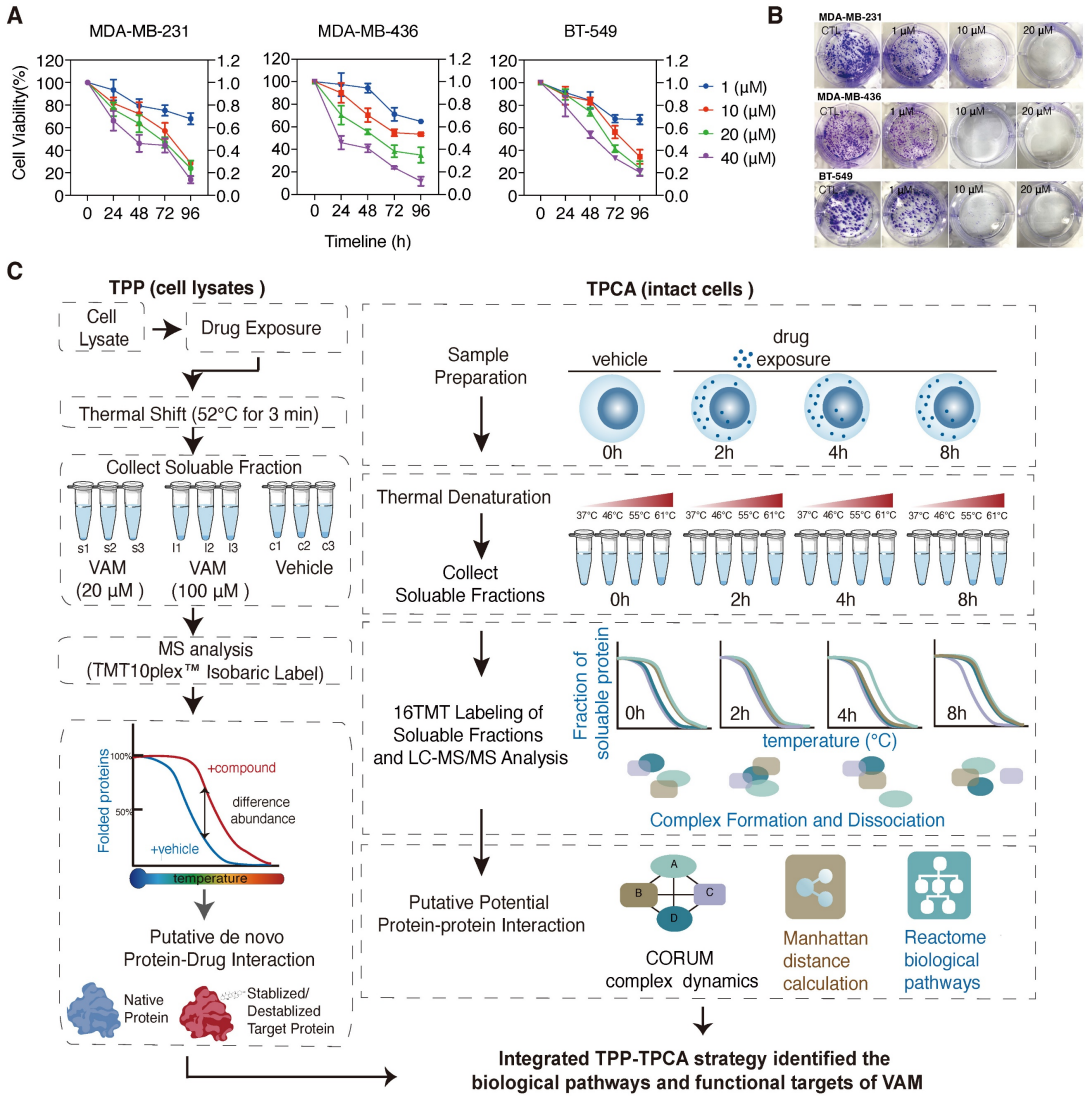
** Schematic illustration of the integrated thermal proteome profiling with thermal proximity co-aggregation (TPP-TPAC) strategy for VAM target identification. (A)** Human breast cancer cell lines MDA-MB-231, MDA-MB-436, and BT-549 were given indicated concentrations (1-40 μM) of VAM for 24, 48, 72, and 96 hours. The CCK-8 test was used to determine cell viability. **(B)** Colony formation assays were performed for 12 days. **(C)** Schematic illustration of the integrated TPP with TPCA strategy to identify biological pathways and potential targets of VAM. TPP experiments were conducted using MDA-MB-231 cell lysates. The samples were separated into 9 identical fractions, subjected to DMSO, low dose VAM and high dose VAM treatment, and then exposed to the thermal challenge (52 °C, 3 min) to denature and irreversibly precipitate thermo-unstable proteins. TPCA experiments were conducted using intact MDA-MB-231 cells. Cells were treated with VAM for 0h, 2h, 4h, 8h and then aliquoted and heated at different temperatures (37°C, 46°C, 55°C, 61°C). SISPROT method 67 was used for MS sample preparation. Peptide abundance was quantified through multiplexed MS and Manhattan distance was used for assessing protein-protein interaction.

Recently, VAM (CAS Number: 60-70-8) was reported to exhibit anti-proliferative and anti-migration activities on broad types of cancer cells 18, 19, 25, 26. Similarly, we observed that VAM exhibits anti-proliferative effects across three human triple-negative breast cancer (TNBC) cell lines in a time- and concentration-dependent manners (**Figure 1A**). VAM also dramatically inhibited colony formation of cells at around 1 μM onward (**Figure 1B**). Previous study demonstrated that this efficiency was related to Hedgehog (Hh) signaling 18.

Here, we integrated iTSA 27, a simplified single-temperature format of TPP, and TPCA to characterize the MoA behind the anti-cancer property of VAM, as illustrated in **Figure 1C**. Our initial step involved the identification of direct ligand-binding proteins that may contribute to the biological activity of VAM. We performed iTSA on MDA-MB-231 cell lysates treated with vehicle and two concentrations of VAM (20 uM and 100 uM). iTSA was performed using cell lysates to maximize the identification of direct binding targets. Out of 5170 proteins detected, we quantified 4242 proteins with at least two peptides (**Figure S1B**) and observed highly correlated protein solubilities across three replicates indicating high reproducibility (**Figure S1A and S1C**). ANOVA analysis revealed 68 ligand-binding proteins with significant differences in thermal stability (adjust p < 0.05) in the presence of VAM (**Figure S1D**).

Next, we performed TPCA on intact cells that were incubated with VAM for 0, 2, 4, and 8 hours to characterize downstream protein complexes and pathways. We used a simplified version of TPCA with improved data analysis algorithms, which involved incubating cells at 37°C, 46°C, 55°C, and 61°C (**Figure 1C**), and allowed us to analyze samples from all four time points concurrently using TMT16 reagents. This method maximized the coherence of detected proteins and reduced runtime batch effects. Data from three biological replicates with high Pearson's correlation were merged for subsequent analysis (**Figure S1E and S1F**).

### 2.2 TPP-TPCA generated a global view of putative targets and the biological process of VAM

**Figure 2:**
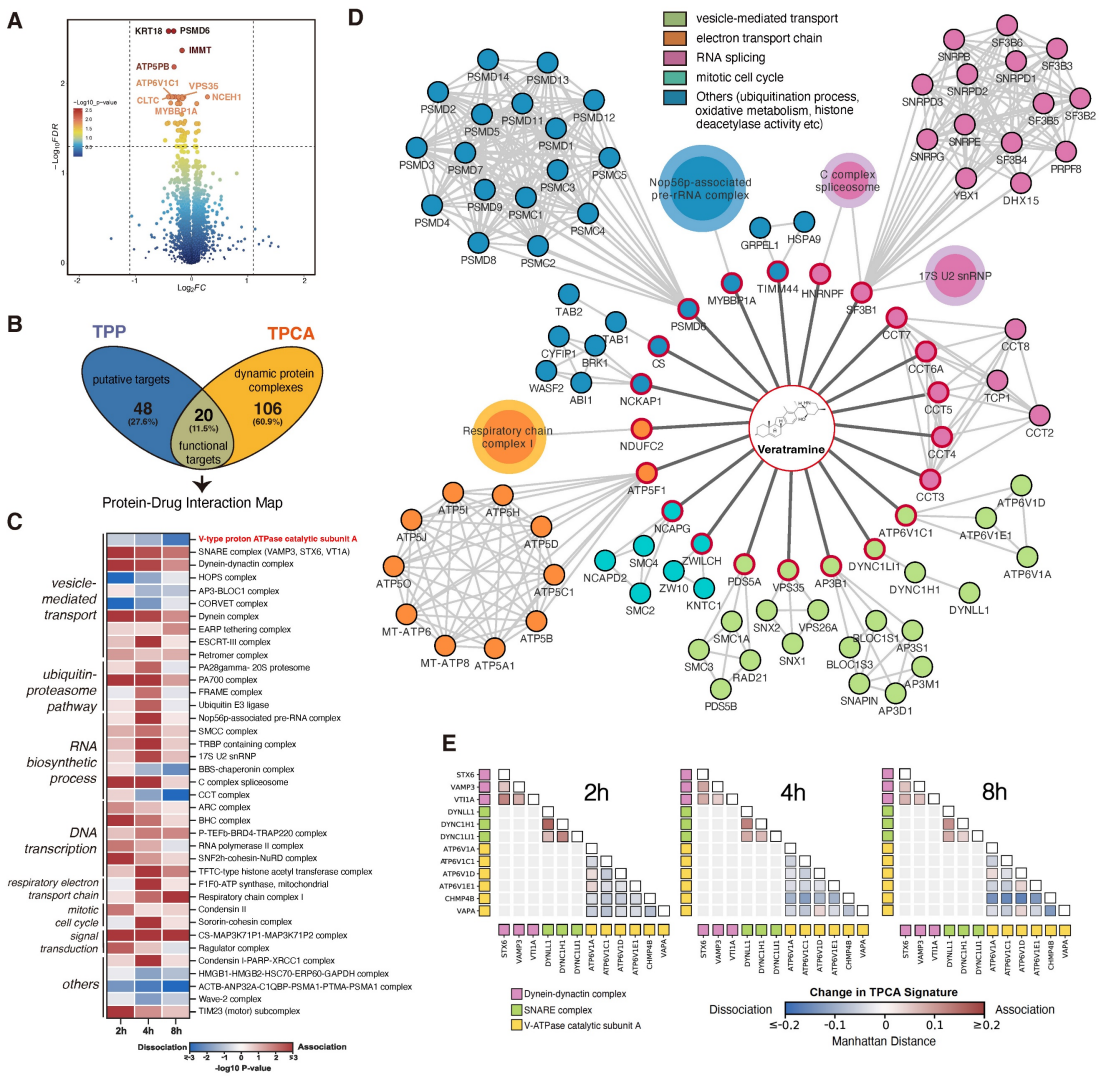
** A global view of binding proteins and perturbed protein complexes of VAM. (A)** Volcano plot of TPP demonstrated the down-regulated and up-regulated proteins as putative targets. Adjust p-value ≤ 0.05. **(B)** Venn plot of overlapping TPP and TPCA. **(C)** Heatmap of modulated complexes and related biological pathways during VAM exposure. **(D)** According to (B), 20 functional targets and 19 convergent/divergent complexes were displayed. **(E)** Distance correlation matrix of any paired subunits in Dynein-dynactin complex, SNARE complex, and V-ATPase complex based on their concatenated abundance-stability profiles.

TPP analysis was performed to identify direct drug-ligand binding, revealing 68 proteins with significant alterations. Notably, most of these proteins displayed decreased solubility, including PSMD6, IMMT, ATP5PB, ATP6V1C1, CLTC, and NCEH1 (**Figure 2A**). To investigate the potential impact of the putative targets on protein complexes and pathways, we employed TPCA, a method that enables quantification of the relative assembly state of protein complexes. An increase or decrease in overall similarity in solubility among subunits indicates an increasingly assembled (convergent) or disassociated (divergent) protein complex, respectively. A total of 3550 human protein complexes annotated in the CORUM database were analyzed with TPCA, revealing 126 complexes that were significantly modulated. More convergent protein complexes were observed at the 2h and 4h timepoints while more divergent protein complexes were observed at the 8h timepoint (**Figure S1G and Table S1**).

TPP-TPCA strategy revealed a direct linkage between 20 functional targets to downstream perturbed protein complexes (**Figure 2B and 2D**). These putative targets and protein complexes identified were found to be involved in vesicle-mediated transport, protein folding, electron transport chain, etc. Among these, we observed multiple modulated protein complexes associated with vesicle-mediated transport, prompting further investigation (**Figure 2C**). Notably, the V-ATPase 23, 28, which was a key regulator of the endo-lysosome system's pH, displayed divergent TPCA signatures at 4h and 8h (**Figure 2E**). We also observed class C core vacuole/endosome tethering (CORVET) complex and HOPS complex which were coordinators of endosome and lysosome fusion 29, 30, also displayed divergent TPCA signatures (**Figure S1H**). On the other hand, the SNARE and Dynein-dynactin complexes 31-33, related to autophagy and endo-lysosomal trafficking, showed convergent phenotype (**Figure 2E**). Based on these observations, we hypothesize that V-ATPase, pH sensor of the endo-lysosome system, may play a critical role in molecule MoA.

### 2.3 VAM directly bound to the ATP6V1C1 and impaired the V-ATPase activity

**Figure 3:**
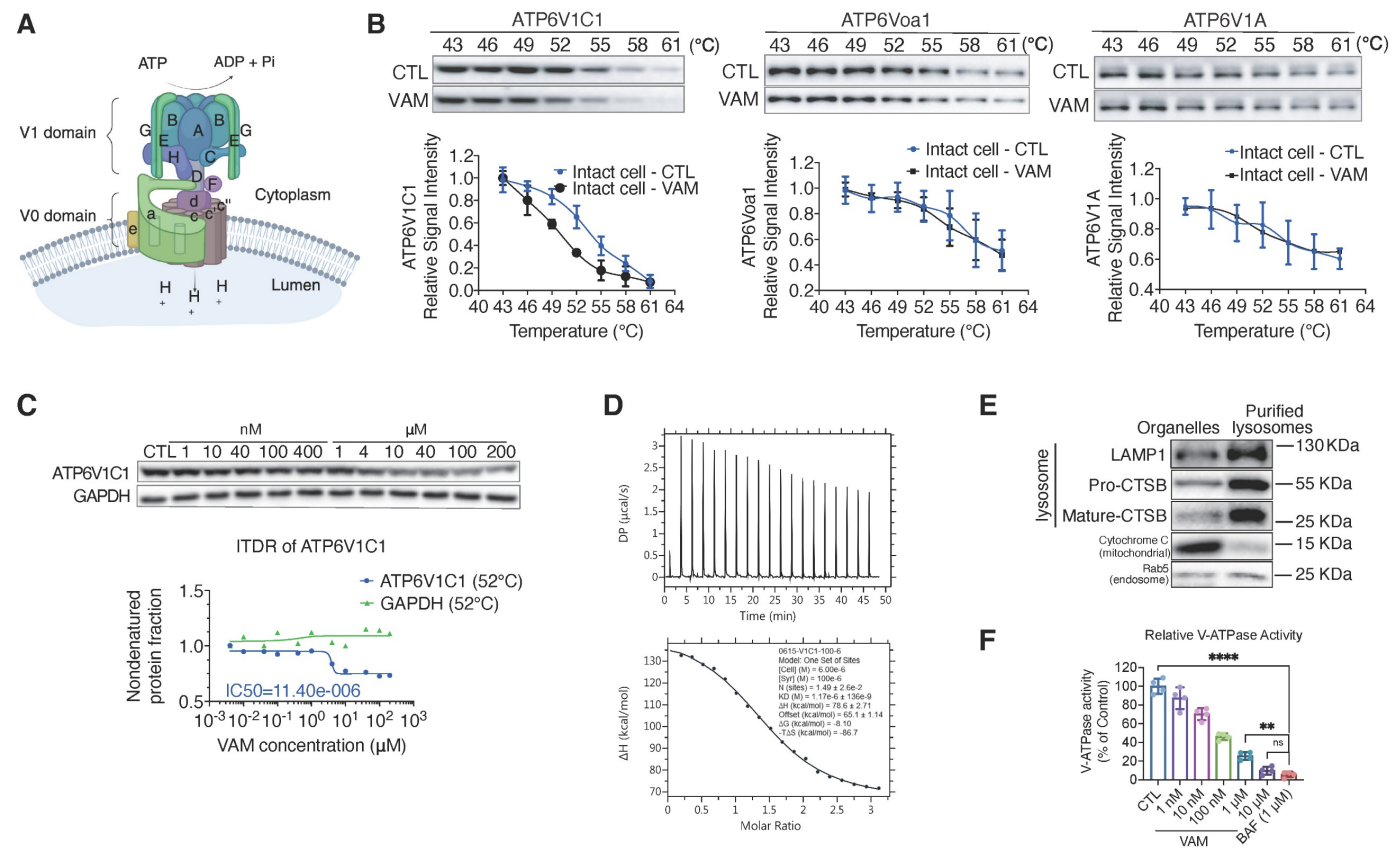
** VAM directly bound to the V-ATPase subunit V1C1 and impaired the V-ATPase activity. (A)** The structure of V-ATPase (created with BioRender). **(B)** Thermal stabilization of ATP6V1C1, ATP6V0a1, ATP6V1A after 10 μM VAM treatment in intact cells and quantification of western blotting (n=3). **(C)** Dose-dependent destabilization of ATP6V1C1 by VAM was evaluated by ITDRF. **(D)** ITC binding curve illustrated titration of VAM (100 μM) into purified ATP6V1C1 (6 μM). The panels show the integrated curve fit to one set of sites (K_D_=1.17 μM). **(E)** Rab5, CTSB, LAMP1, and cytochrome C levels in the purified lysosomal fraction and organelle pellet were examined by western blotting. **(F)** Assay of V-ATPase activity using purified lysosome. The dose of VAM varied from 1 nM to 10 μM and BAF was used as positive control (n=4). One-way analysis of variance (ANOVA) and Turkey post hoc tests were used for the statistical study.

V-ATPase is a multi-subunit complex consisting of a peripheral V1 domain responsible for ATP hydrolysis and a membrane integral V0 domain that translocates protons. Its composition is shown in **Figure 3A** 34. Following the identification of V-ATPase as a key perturbed protein complex during VAM treatment through TPP-TPCA assay (**Figure 2A, 2E**), we proceeded to validate ATP6V1C1, a subunit of V-ATPase. This subunit exhibited significant thermal destabilization as identified from TPP (**Table S1**). To better understand how VAM could generally affect thermal stability of V-ATPase, we conducted cellular thermal shift assay (CETSA) of ATP6V1C1, ATP6V0a1 (a component of V0 domain), and ATP6V1A (a component of V1 domain). Western blotting showed a striking shift of the melting curve denoted by the direct interaction between VAM and ATP6V1C1, while ATP6V0a1, ATP6V1A, and GAPDH showed proximate melting curves (**Figure 3B and S2A**). The abundance of ATP6V1C1 determined by MS analysis showed high consistency with western blotting (**Figure S2B**). Subsequently, an isothermal dose-response fingerprinting based on CETSA (ITDRF_CETSA_) experiment demonstrated a destabilization of ATP6V1C1 by VAM with a half-maximal destabilization at 11.4 μM (**Figure 3C**). Isothermal titration calorimetry (ITC) was conducted to ascertain the binding constant (K_D_ value), revealing a direct interaction between ATP6V1C1 and VAM with a K_D_ value of 1.17 μM (**Figure 3D**). To assess the impact of destabilization of ATP6V1C1 on V-ATPase activity, we isolated lysosomal fractions from rat liver using sucrose density gradient centrifugation and probed with lysosomal associated membrane protein 1 (LAMP1), cathepsin B (CTSB), cytochrome C, and Ras-related protein Rab-5A (Rab5) antibodies to confirm purification process and exclude interference from mitochondria and endosomes (**Figure 3E**). Subsequently, we performed an ATP/NADH (NADPH)-coupled assay, a conventional method to measure ATPase activity 35. Bafilomycin A1 (BAF), a classical V-ATPase inhibitor, was used as the positive control. We found that VAM exerted a dose-dependent inhibitory effect on V-ATPase activity, which was similar to that of BAF, with an approximate 90.24% inhibition at a concentration of 10 μM (**Figure 3F**). Given the potential toxicity of lysosomal inhibition, we assessed cell viability by comparing the effects of BAF and VAM. We found both BAF and VAM induced substantial cytotoxic effects (**Figure S2C-D**). In addition to the inhibition of cancer cell viability, VAM also exhibited mild toxicity to normal cells (**Figure S2E**). Notably, VAM demonstrated broad-spectrum inhibitory effects across different breast cancer subtypes (**Figure S2F-G**). Previous studies reported that ATP6V1C1 participated in the assembly of the V0 and V1 domains 36-39. Therefore, we analyzed the impact of VAM on V0/V1 assembly and V-ATPase activity. We observed that VAM had no effect on the co-location of the V1 domain and lysosomes (**Figure S2H**) and did not hinder the interaction between ATP6V1A, ATP6V0a1 and ATP6V1C1, indicating that VAM did not disrupt the intact structure of V-ATPase (**Figure S2I**). Collectively, these data indicated that VAM directly bound to ATP6V1C1 and inhibited the V-ATPase, without inducing the disassembly of V-ATPase.

### 2.4 VAM impaired lysosome acidification and lysosomal enzyme activity

**Figure 4:**
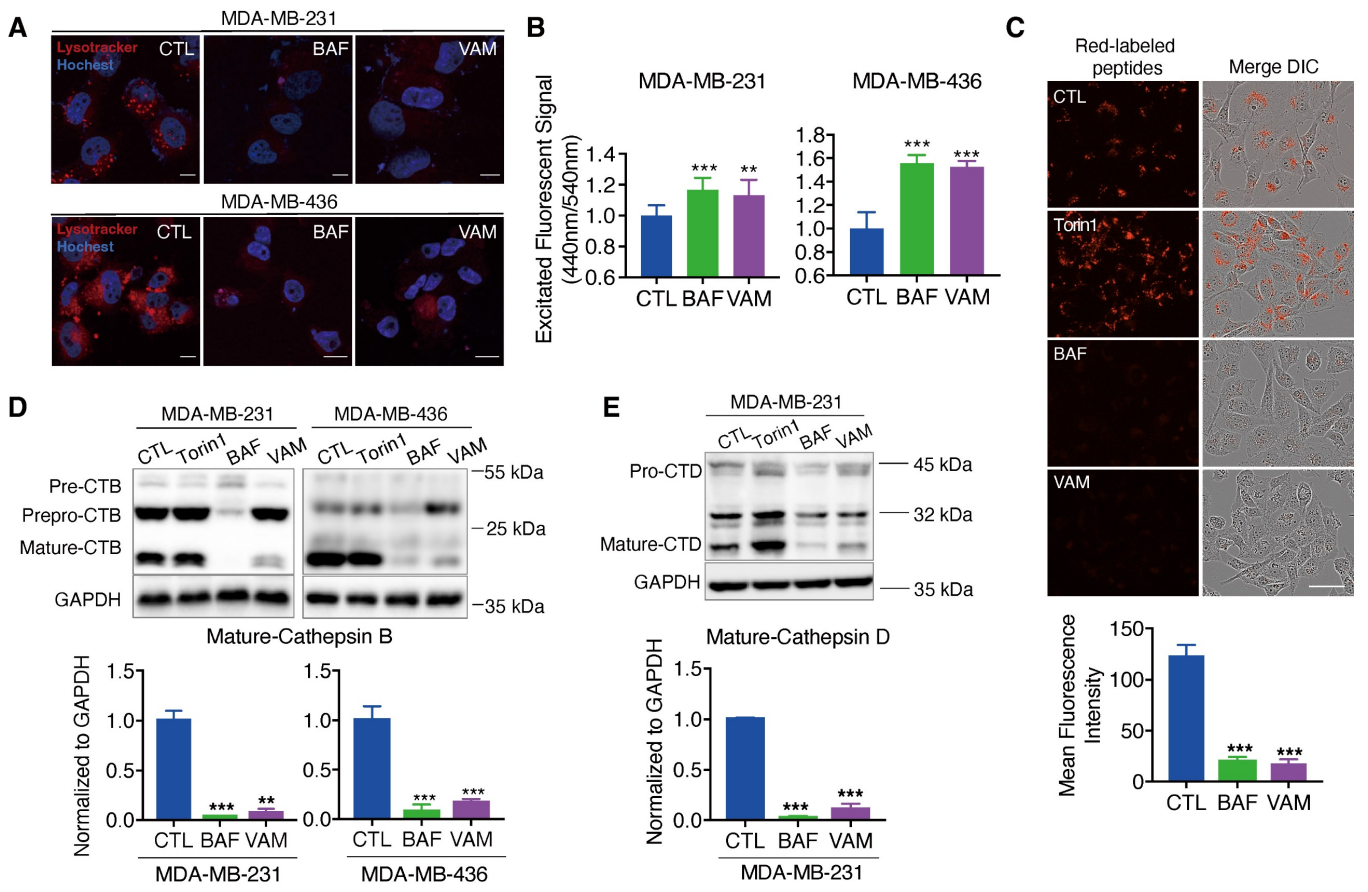
** VAM impaired lysosome acidification and lysosomal enzyme activity. (A)** The cells were exposed to 10 μM VAM, 1 μM BAF, or DMSO for 24 hours. The lysosomes were tagged with LysoTracker™ Red DND-99 for 30 min, fixed, and then stained with Hoechst.** (B)** The pH assessment was conducted using LysoSensor™ Yellow/Blue DND-160. The excited fluorescence signal at 440/540 nm was determined (n=6). **(C)** Serum-free DMEM was used to pre-incubate the cells with 10 μg/ml DQ™ Red BSA for 30 minutes. Subsequently, the cells were exposed to a concentration of 10 μM VAM, 100 nM Torin1, 1 μM BAF, or DMSO for a duration of 6 hours in complete medium. Scale bar: 50 μm. **(D, E)** Cells were exposed to VAM, BAF, Torin1, or DMSO for 24 h. Western blotting to determine the levels of CTSB and CTSD (n=3).

The V-ATPase is a proton pump responsible for sustaining the acidic conditions of lysosomes and other enclosed compartments by actively transporting protons into the lumen 40-43. So, we examined whether V-ATPase inhibition by VAM will impact lysosome acidification and cargo degradation. First, acidic organelles in live cells were tracked using the red-fluorescent dye, LysoTracke^TM^ Red DND-99. As seen in **Figure 4A**, cells almost completely lost red fluorescence following VAM treatment, signifying the absence of acidic organelles. Lysosome pH was determined by a ratio-metric probe, LysoSensor^TM^ Yellow/Blue DND-160. **Figure 4B** demonstrated that the lysosome was alkalized after VAM treatment, similar to the effects of BAF.

Besides, the acidity environment of lysosomes is necessary for cathepsins maturation and cargo degradation. So, we employed the molecular probe DQ™ Red BSA, which can be hydrolyzed into a single dye-labeled peptide by acid protease and produce a bright fluorescent product in lysosomal acidic environment. A notable reduction in red fluorescence was observed in groups treated with BAF and VAM, indicating impaired lysosomal proteolytic activity (**Figure 4C**). Then, the protein level of lysosomal proteases, cathepsins B (CTSB) and D (CTSD), which were responsible for driving proteolytic degradation was analyzed. CTSB and CTSD are initially expressed as inactive precursors, which are activated under an acidic environment through sequential enzymatic cleavage 44-46. We found that VAM dramatically reduced the mature form of CTSB and CTSD, meaning that the cathepsins maturation process in lysosomes was impeded (**Figure 4D and 4E**). Overall, VAM blocked the acidification of the lysosome and proteolytic activity through binding with ATP6V1C1.

### 2.5 VAM blocked the maturation of autophagosomes in TNBC

**Figure 5:**
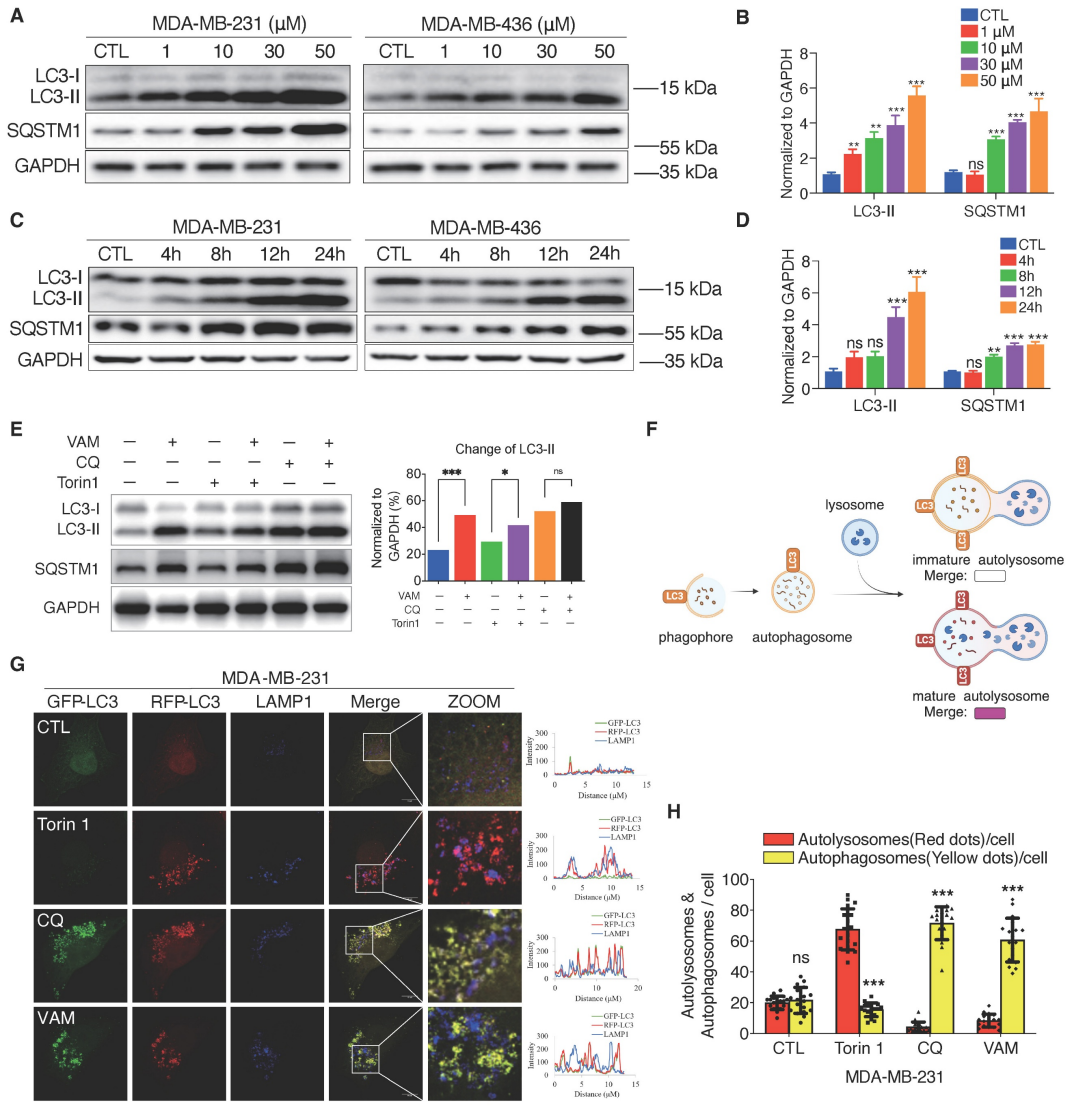
** VAM blocked autophagy-lysosome pathway. (A, B)** LC3B-II and SQSTM1 levels were analyzed using Western blotting. Corresponding quantification was assessed (n=3).** (C, D)** LC3B-II and SQSTM1 levels were analyzed in cells treated with/without VAM at specified time points (0, 4, 8, 12, 24 h). Corresponding quantification was assessed (n=3). **(E)** Incubate MDA-MB-231 cells with 10 μM VAM, 0.5 μM Torin 1, 20 μM CQ separately or in combination for 24 hours. Analyze the expression of LC3B-II through Western blotting (n=3). **(F)** Diagram showing mature autolysosomes and non-fused autophagosomes by analyzing the co-distribution of RFP-GFP-LC3 and lysosomes (created by BioRender). **(G)** RFP-GFP-LC3 MDA-MB-231 cells were treated with VAM, Torin1, CQ or DMSO for 24 h and immuno-stained with anti-LAMP1 antibody. Scale bar: 10 μm. **(H)** Quantification of autophagosomes and autolysosomes was performed by counting the yellow (non-colocalized) and red (colocalized) puncta (n=20).

Autophagy is a self-digesting process responsible for the removal of misfolded proteins, pathogens, and damaged organelles. These components are engulfed to form the autophagosomes and then delivered to the lysosomes 47. Therefore, lysosomal acidification is required for the degradation of autophagic cargo 40. We next tested whether VAM affected autophagy flux. During the autophagy process, the microtubule-associated protein 1 light chain 3 (LC3) can undergo a transformation from LC3-I to its lipid equivalent, LC3-II 48. LC3 and p62/SQSTM1, a cargo adaptor protein, are widely-used indicators for autophagy monitoring. First, we assessed autophagosome formation and found that VAM significantly increased LC3-II levels in both dose-dependent (**Figures 5A, 5B**) and time-dependent manners (**Figures 5C, 5D**). As the substrate of autophagy, SQSTM1 levels increased during VAM treatment suggesting that the substrate degradation by autophagy was impeded.

Chloroquine (CQ), an inhibitor of autophagic flux was used as a positive control 49, 50. Then, we observed that VAM was unable to further increase the LC3-II level in the presence of CQ, indicating that VAM acted similarly to CQ on the autolysosome degradation (**Figure 5E**). To verify this conjecture, we conducted quantitative colocalization analyses of RFP-GFP-LC3B and LAMP1, shown in the diagram in **Figure 5F.** The RFP-GFP-LC3B reporter is a biosensor that incorporates a pH-insensitive RFP protein and a pH-sensitive GFP protein. The fluorescence of GFP will quench in an acidic environment. Therefore, the red/green fluorescence ratio could differentiate immature autophagosomes and mature autolysosomes. Torin1, a mTOR inhibitor, can activate the autophagy and lysosome biogenesis 51, 52. We noticed the high intensity of red fluorescence in Torin1 treated cells. Instead, the yellow fluorescence intensity in cells treated with CQ and VAM showed a notable rise (**Figure 5G and 5H**), denoting the accumulation of immature autophagosomes. Co-localization analysis of LC3 and lysosome in **Figure 5G** also showed the reduced autophagosome and lysosome colocalization in VAM group (**figure S3A**). In summary, VAM disrupted the autophagy process by impairing lysosomal function, thereby inhibiting the fusion of autophagosomes and lysosomes.

### 2.6 VAM enhanced the therapeutic efficacy of doxorubicin

**Figure 6:**
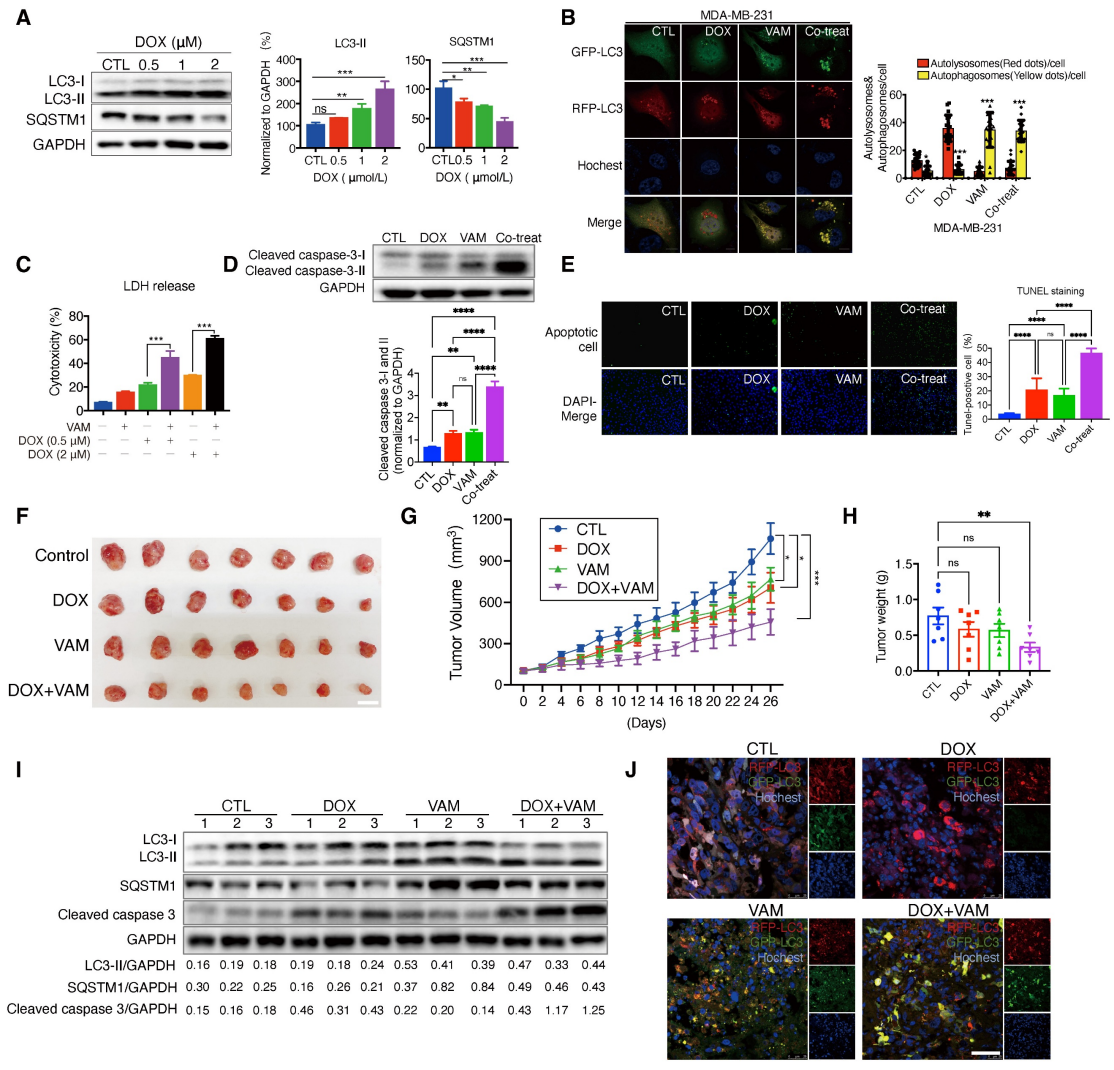
** VAM enhanced anti-tumor effects of DOX *in vivo* and *in vitro*. (A)** MDA-MB-231 cells treated with DOX (0.5, 1, 2 μM) for 24 h. The levels of cytosolic LC3 and SQSTM1 were assessed by western blotting (n=3). **(B)** MDA-MB-231 cells which were genetically modified to express GFP-RFP-LC3 were treated with DOX (1 μM), VAM (10 μM), DOX combination with VAM, or DMSO. The fluorescence images were scanned via Leica TCS SP8 Confocal System. Scale bar: 10 μm. **(C)** MDA-MB-231 cells were exposed to DOX at concentrations of 0.5, and 2 μM, either with or without VAM for 48 h. Cytotoxicity was evaluated using LDH assays (n=4). **(D)** Western blotting analysis of cleaved-caspase 3 (n=3). **(E)** In Situ Cell Death Detection Kit was used to identify apoptosis-positive cells. Scale bar: 100 μm.** (F)** The images show tumors at the time of sacrifice from the vehicle, DOX (2 mg/kg), VAM (3 mg/kg), and combination treatment groups (scale bar: 1 cm). **(G)** Calculation of the average tumor volume. Error bars represent means ± SEM.** (H)** Tumor weight measurement following the sacrifice. Error bars represent means ± SEM. **(I)** Western blotting analysis of LC3-II, SQSTM1 and cleaved caspase 3 in tumor tissues (n = 3).** (J)** Tumor tissues were sectioned and stained with Hoechst. The fluorescent images were captured using confocal microscope (scale bar: 50 μm).

The regulation of autophagy plays a significant role in tumor chemoresistance 53, 54. Therefore, we investigated whether VAM could enhance anti-tumor efficiency when combined with the anti-cancer agent doxorubicin (DOX). DOX is a topoisomerase 2 inhibitor extensively utilized in cancer chemotherapy. We initially explored whether DOX-induced protective autophagy in breast cancer. As depicted in **Figure 6A**, DOX dose-dependently upregulated LC3-II and degraded SQSTM1, indicating the initiation of protective autophagy. Additionally, we constructed a GFP-RFP-LC3B reporter cell line to monitor autophagic flux, revealing cytoplasmic accumulation of RFP-only puncta following DOX treatment, indicative of a speeded autophagy process. On the contrary, the combination of VAM and DOX markedly impeded autophagosome maturation (**Figure 6B**). To further confirm the synergistic effect of DOX and VAM, we assessed the LDH release, level of cleaved caspase 3 (isoforms I and II), and Tunnel staining in cells treated with VAM, DOX, or their combination. Notably, VAM markedly enhanced the pro-apoptotic efficacy of DOX (**Figures 6C-6E**). We also extended the potential of VAM in combination with paclitaxel and epirubicin, demonstrating a synergistic effect (**Figure S4A**).

Subsequently, we performed xenograft experiments to ascertain whether the augmented chemosensitivity in cancer cells due to VAM could be reproduced *in vivo*. Nude mice were injected with MDA-MB-231 cells that had been genetically modified to express GFP-RFP-LC3. They were then treated with DOX or VAM alone or in combination for 4 weeks. As illustrated in **Figures 6F** and** 6G**, the DOX treatment alone group exhibited a tumor growth inhibition rate of 56.71%, whereas combining VAM and DOX resulted in a considerable reduction of 80.56%. As shown in **Figure 6H**, there was a consistent reduction in tumor weight in the co-treatment group when compared to DOX alone (P=0.0229). Besides, there was no obvious difference of body weight (**Figure S4B**) or discernible organ damage (**Figure S4C**), denoting that the dosage of VAM was tolerant in mice.

Next, we analyzed the autophagic flux and apoptosis levels in xenograft tumor tissues. The results demonstrated that co-treatment of DOX and VAM blocked the autophagy flux and increased the level of cleaved caspase 3 compared with DOX treatment group (**Figure 6I**). Besides, we also observed that DOX-triggered "protective autophagy" was inhibited by VAM, with co-treated mice exhibiting more defective autolysosomes (**Figure 6J**). Overall, these findings indicated that breast cancer cells up-regulated autophagy as a defense mechanism during chemotherapy, and autophagy/lysosome suppression by VAM could render the cancer sensitivity to chemo-drugs *in vitro* and *in vivo*.

### 2.7 Expression level of *atp6v1c1* related to tumor progression

**Figure 7:**
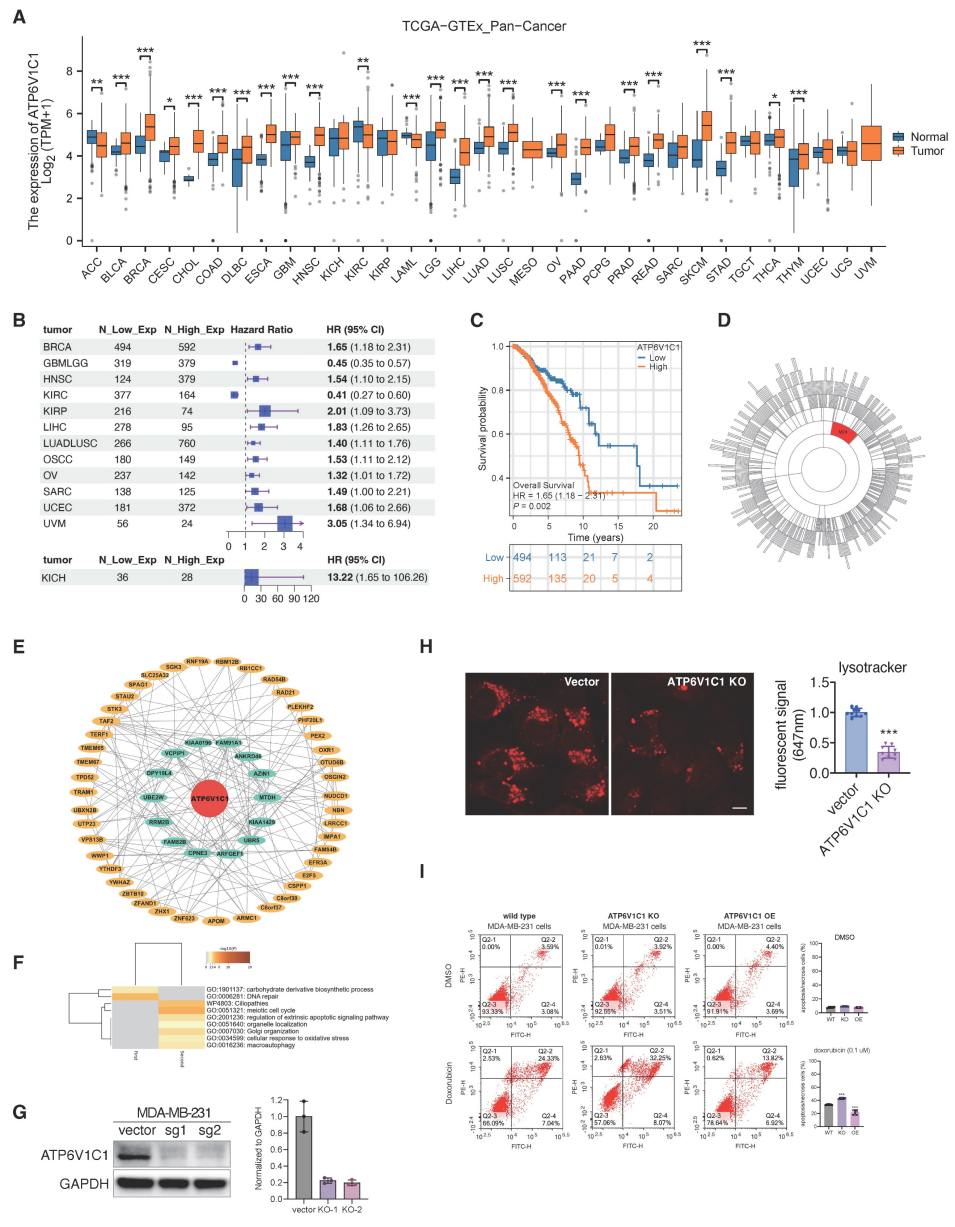
** Expression level of ATP6V1C1 related to tumor progression. (A)** Pan-cancer expression analysis of ATP6V1C1 gene. The expression profile of ATP6V1C1 in 33 common malignant tumors and their corresponding normal tissues. **(B)** Kaplan-Meier Analysis of ATP6V1C1 expression differences across 13 tumor types (HR > 1: higher risk; HR < 1: lower risk). **(C)** Overall survival (OS) comparison between high and low expression of the ATP6V1C1 gene in BRCA. **(D)** ATP6V1C1 is the hub gene of M24 module of BRCA. **(E)** ATP6V1C1 centered two-layer subnetwork in M24 module of BRCA. **(F)** GO analysis of ATP6V1C1 centered two-layer subnetwork. **(G)** Verification of CRISPR KO efficiency of MDA-MB-231 via western blotting.** (H)** Lysotracker staining of ATP6V1C1 KO and parental cells (MDA-MB-231). **(I)** Treatment of parental, ATP6V1C1 KO, and ATP6V1C1 OE cells with/without doxorubicin (0.1 μM) for 72 hours, followed by quantitative analysis of cytometry.

In prior investigation, we observed the targeting of ATP6V1C1 by VAM and demonstrated its anti-tumor efficiency. Thus, we formulated the hypothesis regarding the involvement of ATP6V1C1 in tumor progression. We assessed *atp6v1c1* expression across 33 tumor types and corresponding normal (or adjacent cancer) tissues using TCGA and GTEx datasets. The findings indicate a significant upregulation of ATP6V1C1 in most tumors (n=25), notably in breast invasive carcinoma (BRCA), pancreatic adenocarcinoma (PAAD), and liver hepatocellular carcinoma (LIHC) (**Figure 7A**). Kaplan-Meier (KM) analysis reveals a significant deterioration in the prognosis of patients with high ATP6V1C1 expression, indicated by an elevated hazard ratio (HR) in 11 tumor types (**Figure 7B**). The overall survival curve of breast cancer is detailed in **Figure 7C**. Additional KM curves are provided in **Figure S5A**. Gene co-expression network analysis identified ATP6V1C1 as a hub gene in the M24 module, showing a strong correlation or regulatory effect of ATP6V1C1 (**Figure 7D**). Subsequently, we analyzed the ATP6V1C1-centered two-layer subnetwork in the M24 module of BRCA (**Figure 7E**). Clustering analysis of the proteins in this subnetwork suggests that ATP6V1C1 is involved in metabolic pathways and DNA repair mechanisms, as well as processes related to apoptosis, cell cycle regulation, macroautophagy, and stress responses (**Figure 7F**).

To further elucidate how ATP6V1C1 affects breast cancer progression, we constructed the ATP6V1C1 knockout (KO) cells and discovered that the number of acidic lysosomes in ATP6V1C1 knockout cells was significantly reduced, indicating that ATP6V1C1 was related to lysosome function (**Figure 7G, 7H**). Concurrently, we established an ATP6V1C1 overexpression (OE) cell line to investigate whether differential cytosolic ATP6V1C1 expression levels affect susceptibility to chemotherapy (**Figure S6A**). We observed that ATP6V1C1 KO significantly increased the proportion of early apoptosis cells (annexin V+/PI-, Q2-4) and late-stage of apoptosis/necrotic cells (annexin V+/PI+, Q2-2) compared to the parental cell line (from 24.33% to 32.25%) during doxorubicin treatment. In contrast, ATP6V1C1 OE cells exhibited a marked decrease in late apoptotic/necrotic cells (Annexin V+/PI+, Q2-2) illustrated in **Figure 7I** and **Figure S6B**. Conversely, no significant difference was observed under alpelisib exposure (**Figure S6C**). The probable rationale is that alpelisib is primarily indicated for cases harboring PIK3CA mutations, which are not present in this cell line 55. Collectively, high ATP6V1C1 expression correlates with poor tumor prognosis, and ATP6V1C1 knockout sensitized cells to chemotherapy.

## 3. Discussion

Natural products are rich source of bioactive compounds but dissecting their MoA for downstream optimization remain a challenge. Target identification strategy that does not rely on pre-functionalization of a natural compound is highly desirable as the synthesis routes of natural products are not commonly available. In recent years, TPP has emerged as a popular unbiased, label-free method for target identification circumvent the need for chemical synthesis or derivatization. Nevertheless, the likely multi-targets of natural products pose additional challenge in elucidating their MoA. Here, we pioneered an integrated TPP-TPCA strategy, which links putative targets to downstream perturbed protein complexes, to better characterize the MoA of natural products for drug discovery.

In this study, TPP-TPCA strategy was successfully employed for the identification of MoA of VAM. TPP identified 68 putative targets which were integrated with TPCA data converging to 20 functional targets and 19 target-related dynamic complexes. Notably, we observed modulation of several protein complexes associated with vesicle-mediated transport, including the V-ATPase and Dynein-dynactin complexes which were integral to the autophagy and endo-lysosomal systems. Among them, ATP6V1C1, a critical component of V-ATPase, exhibited the most significant stability changes in response to VAM, so we verified the involvement of ATP6V1C1 in the MoA of VAM. Knockout experiments demonstrated the essential role of ATP6V1C1 in mediating the anti-apoptotic effects of VAM. However, the potential contribution of additional targets to VAM's anti-proliferative effects remains unclear, reflecting the inherent complexity of dissecting the multi-target mechanisms of natural products. Although the TPP-TPCA approach offers a novel and insightful framework for target identification, its reliance on LC/MS quantification poses a limitation, potentially missing low-abundance functional targets.

Recently, V-ATPase has gained increasing attention due to its differential overexpression in tumor cells 56-58, leading to disruptions in intracellular and extracellular pH levels. These disruptions are associated with tumorigenic and metastatic processes 38, 58-60. Here, the TCGA dataset reveals a correlation between elevated ATP6V1C1 expression and the advancement of malignant tumors. Notably, ATP6V1C1, identified as a hub gene in the M24 module, may serve as a potential therapeutic target or diagnostic marker for BRCA.

Through the TPP-TPCA methodology, we have identified, for the first time, a molecule inhibitor of ATP6V1C1 that impacted the activity of V-ATPase. Based on this, we subjected the ATP6V1C1 inhibitor to breast cancer cells and we confirmed that inhibition of autophagy/lysosome by VAM sensitized cancer cells to chemotherapy through blockage of protective autophagy. Finally, these findings shed light on the MoA of natural products and demonstrate the potential of the TPP-TPCA strategy for target identification, and provided evidence that ATP6V1C1 may represent a potential therapeutic target for BRCA via its effect on autophagy/lysosome.

## Materials and Methods

### Cell culture

ATCC acquired the original MDA-MB-231, MDA-MB-436, BT-549, BT-474, HEK293, MCF-7 cells and genetically edited cells were cultured in Dulbecco's modified Eagle's medium (DMEM) supplemented with 10% fetal bovine serum (FBS; Gibco, 10100-147) and 1% penicillin and streptomycin (P/S). HUVEC cells were also purchased from ATCC (PCS-100-013) which were cultured in endothelial cell medium (ECM; ScienCell) supplemented with 10% Fetal Bovine Serum (FBS), and 1% P/S. Cells were cultured at 37°C in a humid 5% CO_2_: 95% air environment.

### Western blotting

Cell proteins were extracted using ice-cold RIPA buffer (Beyotime, P0013) with protease inhibitor cocktail (MedChemExpress, HY-K0010). Proteins were separated according to gel electrophoresis and subsequently transferred onto 0.45 μm PVDF membranes (Bio-Rad, 1704156). Then we blocked the PVDF membranes with TBST (Tris-buffered saline with 0.1% Tween-20) buffer containing 5% (w:v) nonfat milk powder to reduce non-specific binding. The blots were detected with the corresponding primary antibodies and secondary antibody. Blots were visualized using the Pierce ECL kit (Pierce, 32106) and ChemiDoc MP Imaging System (Bio-Rad, 12003154).

### Thermal shift of TPP and TPCA

MDA-MB-231 cells lysates (TPP sample) were subjected to lysis buffer containing 5 mM β-Glycerophosphate, 0.2% n-dodecyl-β-D-maltoside, 10 mM MgCl_2_, 1 mM TCEP, 50 mM HEPES pH 7.5, 0.1 mM Sodium orthovanadate, and EDTA-free protease inhibitor. Cell suspension was subjected to three cycles of flash-freezing in liquid nitrogen and quickly thawing in water. The cell lysate was treated with VAM (20 μM or 100 μM), heated for 3 min at 52 °C, and cooled down to 4 °C for 5 min. Next, the lysates were centrifuged at 14,000 g for 15 minutes at 4 °C. The supernatant was collected, and soluble proteins were quantified with BCA assay. MDA-MB-231 cells (TPCA sample) were exposed to DMSO or VAM for indicated time (0, 2, 4, 8 h). After treatment, intact cells were heated for 3 min at indicated temperature (37°C, 46°C, 55°C, 61°C), cooled down in 4°C for 5 min, and flash-frozen in liquid nitrogen. After centrifugation, the supernatant was collected, and soluble proteins were quantified with BCA assay.

The TPP samples and TPCA samples were digested by SISPROT 61, a spintip-based device, as previously described. The following steps: sample loading, protein reduction, alkylation, digestion, TMT-labelling, and desalting were performed on the SISPROT equipment. Finally, the eluted peptides were lyophilized and then redissolved for LC-MS/MS analysis. The thermal solubility of the protein was assessed by calculating the ratio of its abundance at various temperatures to its abundance at 37°C. To statistically evaluate the TPCA signature, we calculated the mean Manhattan distance (D_avg_) between the melting curves of all pairs of subunits within each protein complex. The average behavior of distinct subunits of the complex was examined, based on Sun et al.'s research 27, to predict convergent or divergent signatures.

### CETSA assay

CETSA were performed using intact cells following established protocols 62. Cells were collected and resuspended in PBS containing a protease inhibitor cocktail at a concentration of 1 mL per 10^7^ cells and incubated with either DMSO or 10 µM VAM for 15 minutes at 37 °C. Cell lysis was achieved by three cycles of freeze-thawing, with 3-minute incubations in liquid nitrogen and in 37 °C water bath between cycles. Cells were then aliquoted for each temperature condition, heated in a Veriti Thermal Cycler (Applied Biosystems) for 3 minutes at the indicated temperature, and cooled for 5 minutes at 4 °C. Samples were centrifuged at 18,000 × g for 20 minutes at 4 °C to pellet protein aggregates, and 60 µL of the soluble fraction was collected for subsequent western blot analysis.

### Recombinant ATP6V1C1 expression and ITC titration assay

Recombinant proteins were synthesized by Detai Bio Company. Briefly, human wild-type and R309/Q363 mutant ATP6V1C1 were sub-cloned into a modified pET30a vector with an N-terminal 6xHis tag. The recombinant wild-type plasmid was introduced into BL21 (DE3) E. coli. The expression of these plasmids was triggered by the addition of 0.1 mM IPTG and the bacteria were incubated at 15 °C for a duration of 16 h. Following collection, the bacteria underwent sonication and purification using a Ni-iminodiacetic acid affinity chromatography (Ni-IDA) system. ITC was conducted at 25°C using the MicroCal PEAQ-ITC instrument. Lyophilized wild-type and mutant ATP6V1C1 proteins were dissolved in sterilized deionized H_2_O containing 2% DMSO for titration. Protein concentration in the cell pool was 6 μM, and 100 μM of VAM was loaded in the syringe. The background data obtained from injecting VAM into the ITC buffer were subtracted before the data analysis.

### Construction of RFP-GFP-LC3 MDA-MB-231 cell line

HEK293T cells were transfected with a mixture of serum-free DMEM, Lipo8000 transfection reagent, pLVX-Puro-mRFP-GFP-LC3, psPAX2, and pMD2.G, following the manufacturer's instructions. Cells were incubated at 37°C with 5% CO₂ for 6 hours, then the medium was replaced with complete DMEM. Virus particles were collected 48 hours post-transfection, filtered through a 0.45-μm PES filter, and used to infect MDA-MB-231 cells. Puromycin (2 μg/mL) was used for selection, and the medium was changed every 2 days until all un-transduced cells were eliminated. The RFP-GFP-LC3 MDA-MB-231 cells were used for both *in vitro* assays and xenograft mouse models.

### CRISPR-Cas9 genome editing

Knockout cell lines were generated using the CRISPR-Cas9 system. LentiCRISPRv2 vectors, which express a single guide RNA (sgRNA), Cas9, and a selection cassette, were obtained from Addgene (#52961). CRISPick was utilized to select candidate sgRNA sequences (https://portals.broadinstitute.org/gppx/crispick/public). As previously described, oligo pairs encoding the 20-nt guide sequences were ligated into LentiCRISPRv2 vectors via BbsI restriction sites 63. Two distinct sgRNA sequences targeting human ATP6V1C1 were used, with a non-targeting (NT) guide as a control. Lentivirus was packaged to transduce MDA-MB-231 cells, which were subsequently selected with 2 μg/mL puromycin. Individual clones were isolated by limiting dilution and allowed to expand for 6-8 weeks. Efficient deletion was confirmed by immunoblotting for cytosolic ATP6V1C1 level. The sgRNA sequences targeting human ATP6V1C1 are as follows:

sgRNA_1: TAAACAAGGTGACATTACAC

sgRNA_2: TAAGAAAGTAGCTCAATACA

sgNT: GCACTACCAGAGCTAACTCA

### Over-expression ATP6V1C1 cell line

ATP6V1C1 overexpression (OE) vector was designed by Obio Technology. Lentivirus was sourced from Obio Technology as well. MDA-MB-231 cells were infected with lentiviral particles and ATP6V1C1 OE clones were selected using puromycin (2 μg/mL).

### Immunofluorescence

RFP-GFP-LC3 MDA-MB-231 cells were cultured in the confocal dishes. When cells reached 60% confluency, they were treated with DMSO, VAM, Torin 1, BAF or CQ for 24 h. The cells were fixed for 10 min using 4% paraformaldehyde. Then blocked the cells using a 2% BSA solution in PBS. Afterwards, the cells reacted with anti-LAMP1 (1:200) antibodies at 4°C for 12h, followed by incubation with secondary antibody, anti-Rabbit Alexa Fluor™ 647 (1:500). Hoechst was used for nuclei labelling. Fluorescence images were captured using a confocal microscope.

### Lysosomal pH measurements

Lysosomal pH was measured by LysoSensor Yellow/Blue DND-160 probe. MDA-MB-231 and MDA-MB-436 cells were seeded in a 96-well black, clear-bottom plate (Thermo, 165305) at a density of 1 × 10⁴ cells per well to minimize fluorescence interference from edge wells. After Torin1, CQ or VAM treatment for 24 h, cells were labeled with 2 µM LysoSensor Yellow/Blue DND-160 dissolved in DMEM without phenol red (31053028, Gibco) for 30 min at 37°C and washed twice using PBS. Subsequently, 100 μL HBSS (Mg^2+^, Ca^2+^) was added to each well for further detection. Fluorescence was measured using a microplate reader (Molecular Devices FlexStation 3) 10-15 minutes after probe removal to minimize the "alkalizing effect." Lysosomal pH changes were calculated based on the ratio of light emitted at 440/540 nm upon excitation at 329/384 nm, respectively (bottom-read).



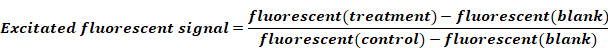



### Incorporation of DQ™ Red BSA conjugates

MDA-MB-231 cells were seeded on 12-well cell culture plates. At 60-70% confluency, cells were incubated with 10 μg/mL DQ™-BSA in serum-free DMEM at 37°C for 30 minutes. Excess probe was removed by washing the cells three times with PBS. Cells were then treated with Torin1, CQ, VAM or DMSO for 6 h. Upon digestion, DQ-Red-BSA releases fragments with excitation and emission maxima at ~590 nm. Fluorescence images were captured using the Incucyte S3 Live-Cell System with the “Phase” and “Red” imaging channels.

### V-ATPase assay

Lysosomes were purified from rat liver following the sucrose density gradient centrifugation 64, 65. Male rat livers were isolated and homogenized in 0.25 M sucrose containing 1 mM EDTA-Na_2_ (pH 7.5), and 10 mM HEPES. The homogenate was filtered and centrifuged at 11,000 g for 20 minutes. A sucrose gradient was prepared by combining a 41% and 20% w/v sucrose solution with post-organellar supernatant. The mixture was then subjected to centrifuged at 112,700 g for 4h. The lysosomal fraction was collected from the interface of the 20% and 41% sucrose solutions and utilized for measuring V-ATPase activity using the ATP/NADPH-coupled assay, as described previously 66. The 1 mL reaction buffer contained 8 U lactate dehydrogenase (LDH), 5 U pyruvate kinase (PK), 5 mM β-mercaptoethanol, 10 mM HEPES-NaOH (pH 7.4), 100 μg/mL BSA, 150 mM KCl, 0.16 mM NADPH, 3 mM phosphoenolpyruvate (PEP), 1 μM ouabain, and 5 mM sodium azide. The lysosomal fraction (10 μg) was incubated with varying concentrations of VAM or BAF for 30 min at RT. The reactions were initiated by adding 15 mM Mg-ATP, and optical density (OD) values were measured using a Microplate Reader. Background values were subtracted. ATP hydrolysis activity = [(initial OD - final OD) for the treatment group] / [(initial OD - final OD) for the control group].

### Xenograft assay

Each nude mouse (4-5 weeks) was injected with 2 × 10^6^ RFP-GFP-LC3 MDA-MB-231 cells, which were suspended in serum-free DMEM. The inoculation was done in the left axilla. The tumor volume was calculated using the following formula: Volume = (longest diameter) × (shortest diameter)^2^/2. The volume ranging from 80 to 120 mm^3^ was deemed a successful xenograft model. We injected VAM (3 mg/kg, every 2 days) or doxorubicin (2 mg/kg, every 6 days) dissolved in 0.9% sodium chloride intraperitoneally into mice alone or together (n = 7). Measurements of tumor size and body weight are taken at two-day intervals. After 26 days of treatment, the mice were euthanized. Organs and tumors were isolated, and either fixed in paraformaldehyde or snap-frozen at -80°C for subsequent analysis.

### Annexin-V/propidium iodide (PI) dual staining assay

A quantitative assessment of apoptosis cells was performed using the Annexin V-FITC Apoptosis Detection Kit (Beyotime Biotechnology, C1062M). In short, the WT, KO, and OE MDA-MB-231 cells were cultured in a 6-well cell culture plate and treated with/without doxorubicin. Then cells were collected, washed with cold PBS, and resuspended in binding buffer (1 × 10^6^ cells/mL). After 100 µL of cells was transferred to a tube, added 5 µL of FITC-conjugated Annexin V (Annexin V-FITC) and 2 µL of PI and incubated for 15 min at RT in the dark. The stained cells were analyzed by the flow cytometer (BD LSR Fortessa™ Flow Cytometer). Data of 10,000 cells were collected in each data file. Four different populations of cells were easily distinguished: un-labelled (viable cells), Annexin V+ (early apoptotic), PI+ (necrotic), and Annexin V+/PI+ positive (late apoptotic/necrotic cells). The cells in each quadrant were determined.

### Colony formation assay

MDA-MB-231, MDA-MB-436, BT-549 cells were seeded in 6 well plate at a density of 200 cells in each well. And treated with VAM as indicated concentrations (1, 10, 20 μM) for 12 days. Medium was replaced every 3 days. Next, cell colony formations were gently washed with PBS and fixed with 4% PFA for 15 min at room temperature. Colonies were stained with 0.5% crystal violet for 20 min at room temperature. The stain was then carefully washed off with running water and dried. Each colony in the wells was imaged for analysis.

### LDH release assay

MDA-MB-231 cells were cultured in 96-well plates at 1 × 10^4^ cells per well. The cells were treated with 10 μM VAM with/without DOX (0.5/2μM) for 24h. the 96-well plates were centrifuged for 5 min in a plate centrifuge at 300× g. Then, 120 μl of the supernatant from each well was transferred to a new 96-well plate. LDH activity in this supernatant was assayed with a LDH Cytotoxicity Assay Kit (Beyotime, C0016) according to the manufacturer's instructions. The absorbance values in the wells were measured at both 490 and 600 nm.

### TUNEL assay

Cell death was detected using the In Situ Cell Death Detection Kit (Roche Applied Science, 11684795910) according to manufacturer's protocol. 5 × 10^5^ MDA-MB-231 cells were seeded in gelatin-coated 24-well plate. treated with DOX/VAM for 24h. the detection of apoptotic cells was assessed via APO-BrdU™ TUNEL Assay Kit (Thermo, A23210). Sections were fixed and incubated with reaction solution for 1 hr at 37°C in a humidified atmosphere in the dark. Images were acquired with an Olympus IX81 microscope.

### CellTiter-Glo assay

For colorimetric assays, cells were seeded into BeyoGold™ White 96-well cell culture plates (Beyotime, FCP968) at a density of 2,000 cells per well. Then the cells were treated with the indicated doses of chemicals for 48 hours. Cell viability was then quantified using the CellTiter-Glo® Luminescent Cell Viability Assay, following the manufacturer's instructions (Promega, G9241).

### Isolation of primary mouse vascular fibroblasts

Primary mouse vascular fibroblasts were isolated from the thoracic aorta of C57BL/6 mice (6-8 weeks old) under sterile conditions. The aorta was excised and transferred to ice-cold PBS containing 1% P/S. It was then minced into small pieces and digested with collagenase I in Dulbecco's Modified Eagle Medium (DMEM) supplemented with 1% P/S at 37°C for 1 hour. The digested tissue was filtered through a 70-µm nylon mesh. The isolated fibroblasts were cultured in DMEM containing 10% heat-inactivated fetal bovine serum (FBS), 1% P/S, and 80 ng/mL of aortic fibroblast growth factor (AFGF).

### Statistical analysis

Each experiment was verified at least 3 replicates using independent samples, and the results were analyzed using GraphPad Prism and presented as mean ± SD or mean ± SEM. Any outliers in data were identified and excluded using GraphPad Prism. One-way analysis of variance (ANOVA) followed by the Turkey as post hoc tests, and two tailed T test followed by Bonferroni correction were conducted using the Graphpad Prism 9. [*P <0.05; **P <0.01; ***P <0.001; and not significant (n.s.)].

## Supplementary Material

Supplementary figures and table.

## Figures and Tables

**Table 1:** Antibody details

Antibodies	Source	Identifier
anti-ATP6V1C1 antibody	Sino Biological	106995-T36
anti-ATP6Voa1 antibody	Proteintech	13828-1-AP
anti-ATP6V1A antibody	Proteintech	17115-1-AP
anti-LC3 antibody	Novus	NB100-2220
anti-SQSTM1 antibody	ABclonal	A11250
anti-Cathepsin D antibody	Santa Cruz	SC-6486
anti-Cathepsin B antibody	Santa Cruz	SC365558
anti-LAMP1 antibody	Cell Signaling	9091
anti-cleaved caspase 3 antibody	Cell Signaling	9661
anti-Cytochrome c antibody	Cell Signaling	4272
anti-Rab5A antibody	Cell Signaling	46449
anti- GAPDH antibody	Cell Signaling	D16H11
anti-rabbit IgG antibody	Cell Signaling	7074
Alexa Fluor Plus 647 Goat anti-Rabbit IgG	Thermo Scientific	A32733

**Table 2:** Chemical and reagent details

Chemicals and reagents		
Veratramine	MedChemExpress	HY-N0837
Doxorubicin	MedChemExpress	HY-15142A
Torin1	LC Laboratories	T-7887
Chloroquine	Sigma	C6628
Baflomycin A1	Sigma	19-148
TMTpro™ 16plex Label Reagent Set	Thermo Scientific	A44520
LysoTracker Red DND-99	Thermo Scientific	M22425
LysoSensor Yellow/Blue DND-99	Thermo Scientific	L7545
Hoechst 33342	Thermo Scientific	H1399
β-mercaptoethanol	Gibco	21985-023
Bovine Serum Albumin	Beyotime Biotechnology	ST023
Paraformaldehyde	Shanghai Sangon Biotech	E672002-0500
DQ-Red BSA	Thermo Scientific	D12051
Annexin V-FITC/PI Apoptosis Detection Kit	Yeasen Biotechnology	40302ES
Lipo8000™ Transfection Reagent	Beyotime	C0533
Protease Inhibitor Cocktail	MedChemExpress	HY-K0010

**Table 3:** Plasmid or recombinant DNA details

Plasmid or recombinant DNA		
lentiCRISPR v2	Addgene	52961
pSLenti-CMV-ATP6V1C1-6xHis-PGK-Puro-WPRE	Obio Technology	N/A
pMXs GFP-LC3-RFP	Addgene	117413
EGFP-LC3	Addgene	11546
pLAMP1-mCherry	Addgene	45147

**Table 4:** Oligonucleotide details

Oligonucleotides		
CRISPR_ATP6V1C1_1 (5ʹ- TAAGAAAGTAGCTCAATACA -3ʹ)	This paper	N/A
CRISPR_ ATP6V1C1_2 (5ʹ- AGAGTATCTCGTCACATTAC -3ʹ)	This paper	N/A

**Table 5:** Recombinant protein details

Recombinant proteins		
ATP6V1C1	Detai Bioscience	N/A
