# Supplementary material for: Integrated thermal proteome and thermal proximity co-aggregation profiling identifies ATP6V1C1 as a novel anti-cancer drug target
Source: Int J Biol Sci. 2025 Apr 28;21(7):3197–213. doi: 10.7150/ijbs.106843 (PMC12080387; doi:10.7150/ijbs.106843)
Supplement: Supplementary file 1 — Supplementary figures and table. [file ijbsv21p3197s1.pdf]

# Supplementary Materials

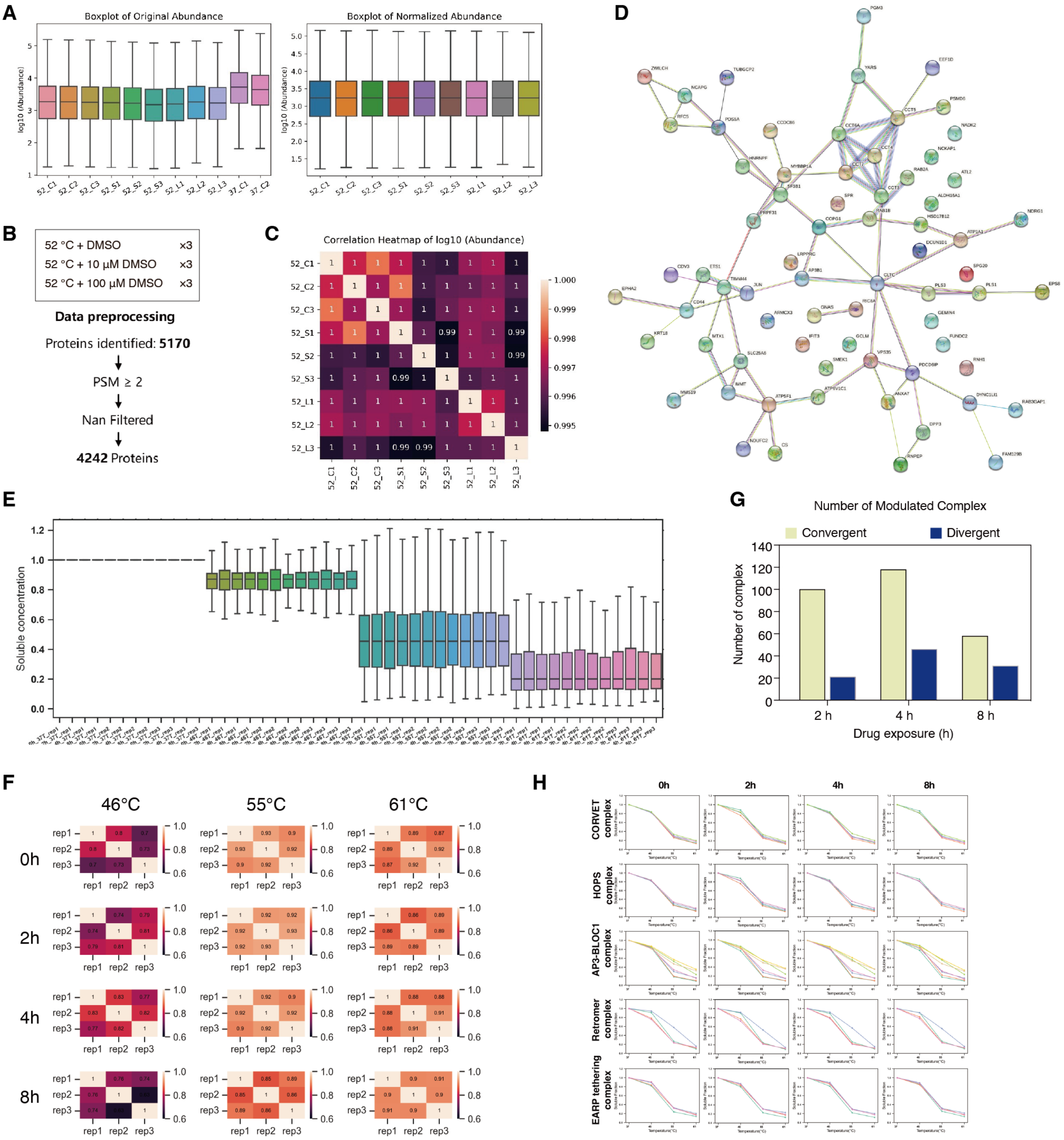


**Figure S1. Quality control and extended results of TPP-TPCA. (A)** The abundance of proteins was normalized by the median value. 9 TMT channels were divided into three groups, control (Ctrl), low dose (S) and high dose (L). **(B)** Workflow of data preprocessing. After PSM filtering and null removal, there were 4242 valid proteins. **(C)** The correlation between abundance in the 9 TMT channels was evaluated using Pearson's correlation coefficient. **(D)** PPI map of 68 differential proteins were analyzed by STRING database. **(E)** The abundance of proteins in TPCA was normalized by the median value. 37, 46, 55, 61 represent the heating 37 degrees, 46 degrees, 55 degrees, 61 degrees, respectively. **(F)** Correlation of peptide abundance in three

replicates was evaluated using Pearson's correlation coefficient. **(G)** The quantity of associated and dissociated complexes observed at various time intervals. **(H)** TPCA profiling of dynamic protein complexes at various time points during VAM exposure (CORVET and HOPS complex, AP3-BLOC1 complex, Retromer complex, EARP tethering complex).

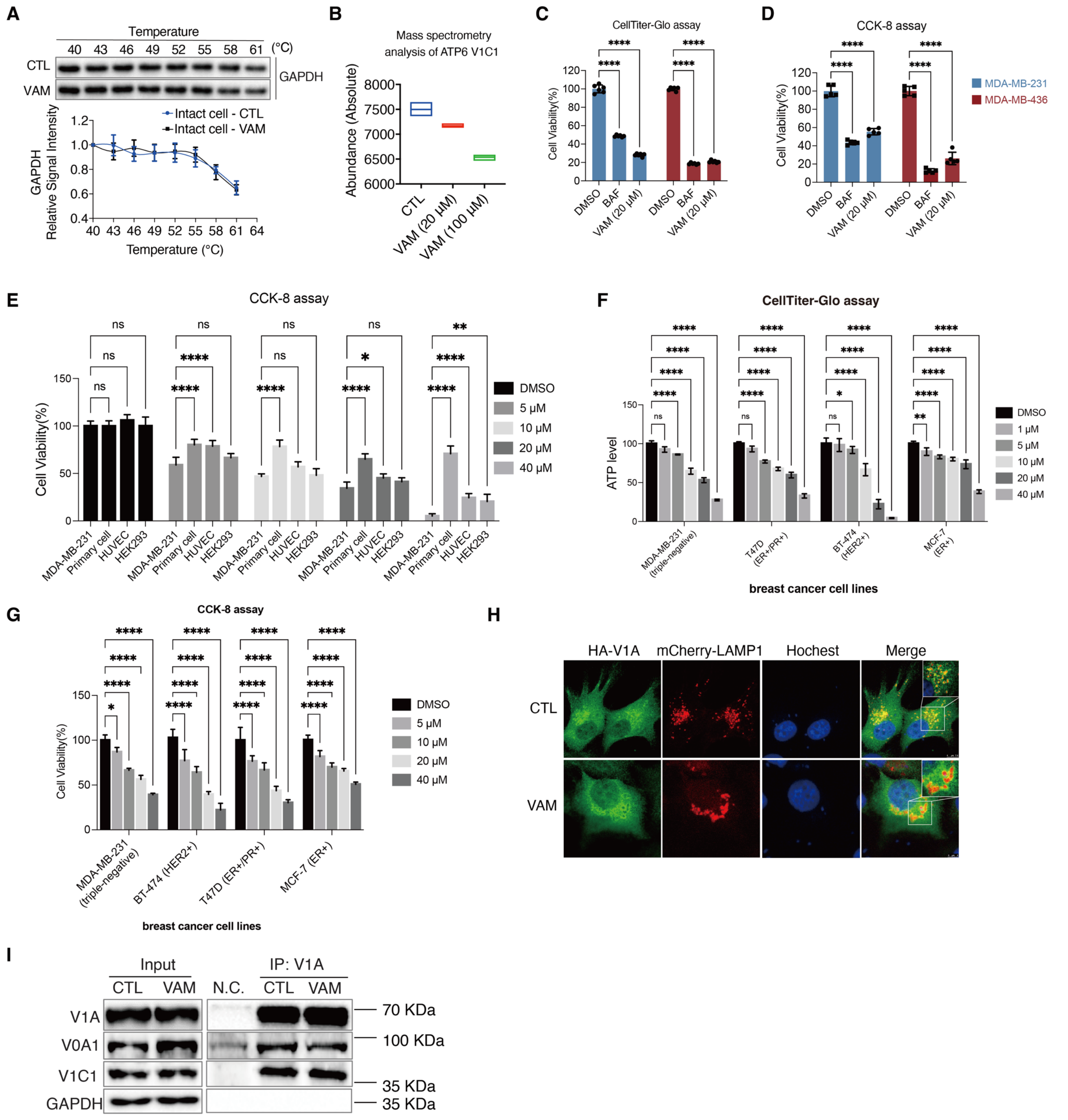

**Figure S2. VAM did not affect V-ATPase assembly. (A)** Melting curve of GAPDH under VAM or DMSO treatment. **(B)** Abundance of ATP6V1C1 in lysates treated with DMSO or VAM was quantified by MS. **(C)** CCK-8 assay assessing the effects of 100 nM BAF and VAM treatment for 48 hours. **(D)** CellTiter-Glo® 2.0 luminescent cell viability assay evaluating the effects of 100 nM BAF and VAM treatment for 48 hours. **(E)** CCK-8 assay was used to evaluate the effects of different doses of VAM treatment on primary cells or non-cancer cell lines for 72 h. **(F)** CellTiter-Glo® 2.0 luminescent cell viability assay was used to evaluate the effect of VAM on different subtypes of breast cancer cells. **(G)** CCK-8 assay was used to evaluate the effect of VAM on the viability of different subtypes of breast cancer cells. **(H)** Co-distribution of V-ATPase peripheral domain (ATP6V1A) and lysosome (LAMP1). **(I)** MDA-MB-231 cells were treated with DMSO or VAM for 24 h. Following treatment, co-immunoprecipitation was conducted using V1A and IgG to evaluate the interaction with ATP6V1C1 and ATP6V0a1.

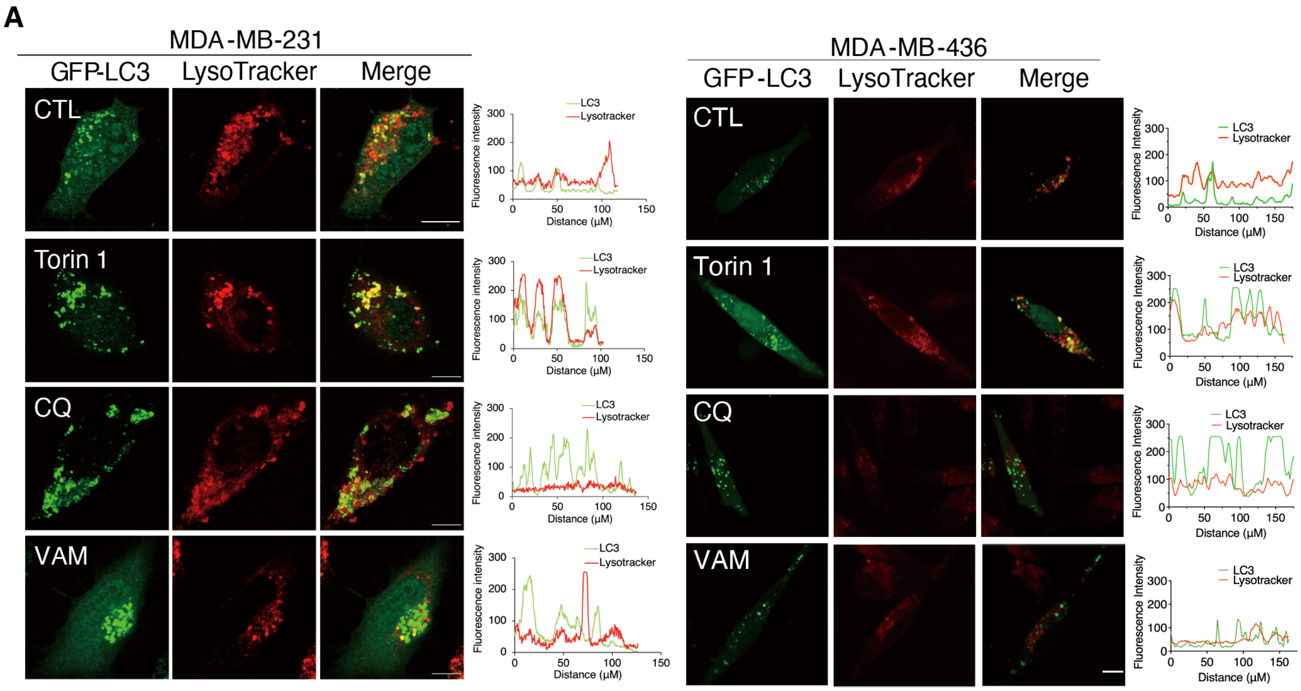


**Figure S3. VAM blocked autophagosome-lysosome fusion. (A)** Colocalization analysis of the autophagosomes and lysosomes. EGFP-LC3 Hela cells were treated with 10 μM VAM, 100 nM Torin1, 30 μM CQ or DMSO for 24 h and stained with LysoTracker Red (50 nM) for 30 min. The fluorescence images of LC3 and lysotracker were scanned via Leica TCS SP8 Confocal Laser Scanning Microscope System. Scale bar: 10 μm.

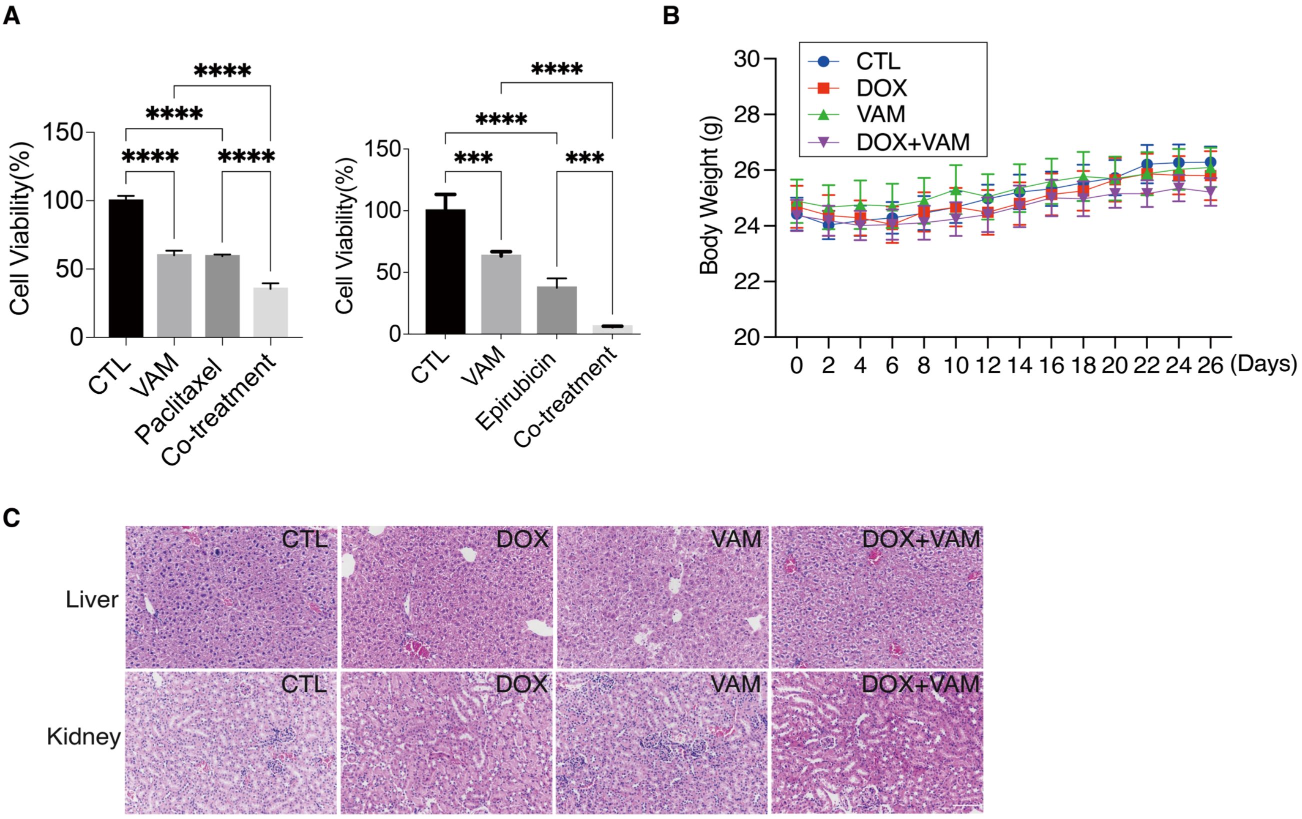


**Figure S4. Toxicity evaluation. (A)** MDA-MB-231 cells were exposed to paclitaxel (10 nM) or epirubicin (0.5 μM) either with or without VAM (20 μM) for 72 h. Cell viability was evaluated using CCK-8 assays (n=4). **(B)** Body weight changes of mice during 26 days of exposure. **(C)** H&E staining assays of the liver and kidneys from all experimental groups (n = 3). Scale bar=100 μm.

A

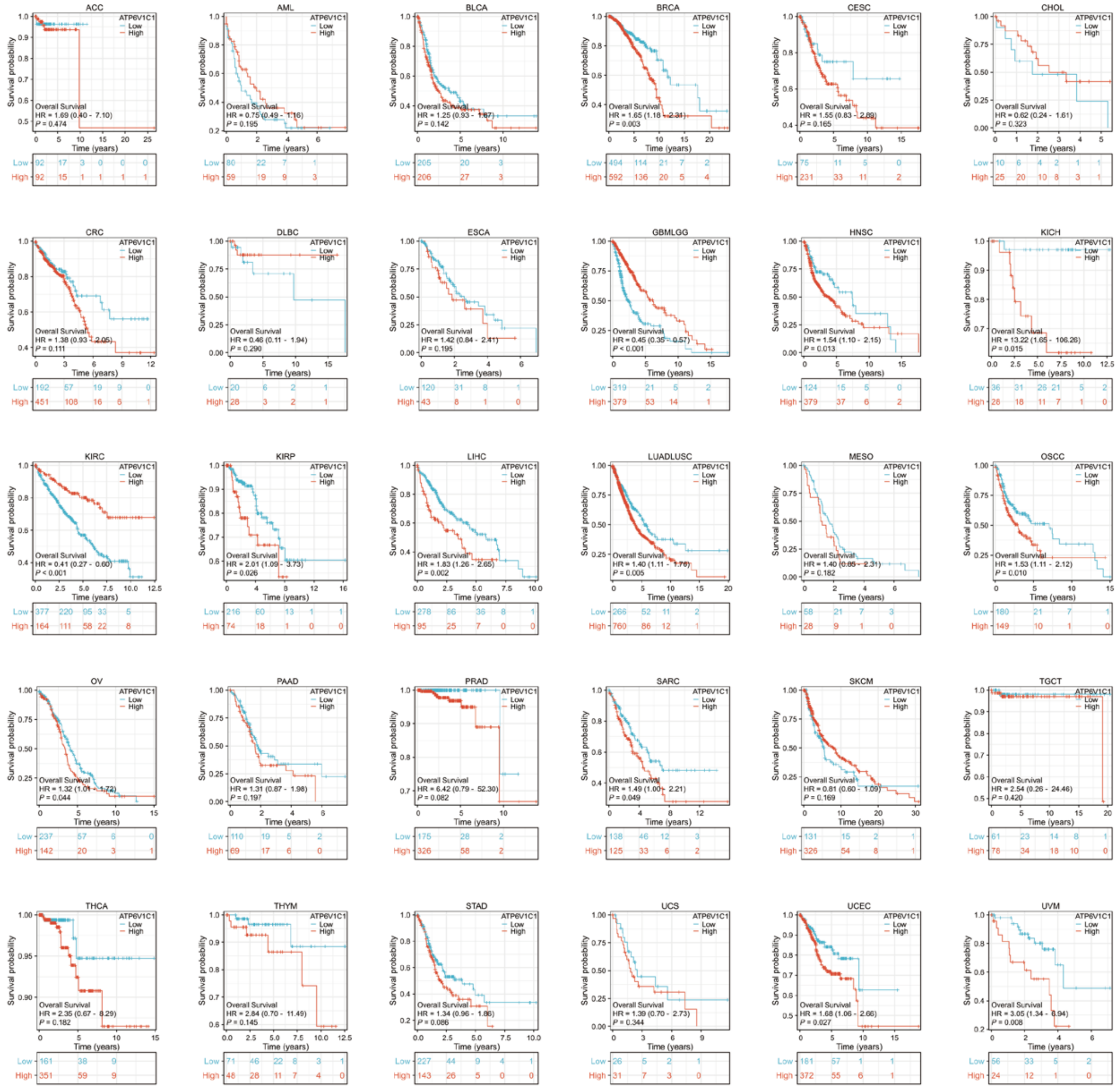

**Figure S5. (A)** Overall survival (OS) comparison between high and low expression of the *atp6v1c1* gene in 33 common malignant tumors.

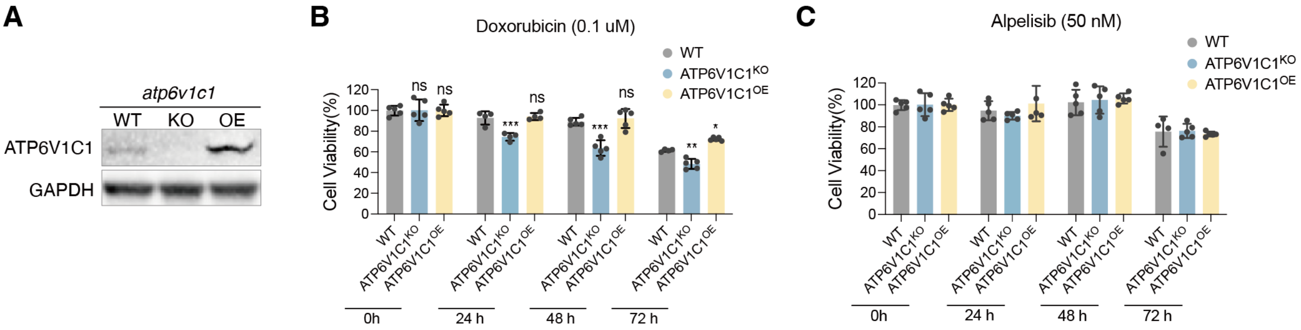


**Figure S6. Influence of cytosolic ATP6V1C1 levels on tumor viability. (A)** Western blot analysis to assess cytosolic ATP6V1C1 levels in WT, KO, and OE MDA-MB-231 cell lines. **(B)** Evaluation of cell viability in ATP6V1C1 WT, KO, and OE cell lines following doxorubicin treatment. **(C)** Assessment of cell viability in ATP6V1C1 WT, KO, and OE cell lines upon paclitaxel treatment (n=5).

**Supplemental Table S1**

**Table S1**: 20 functional targets of VAM identified in TPP-TPCA profiling based on soluble protein abundance.

| Entry | Gene Name | Protein Complex | Adjust P value (DMSO v.s. VAM) |
|---|---|---|---|
| 1 | ATP6V1C1 | V-type proton ATPase catalytic subunit A | 0.0147 |
| 2 | DYNC1LI1 | Dynein-dynactin complex | 0.0427 |
| 3 | AP3B1 | AP3-BLOC1 complex | 0.0490 |
| 4 | VPS35 | Retromer complex | 0.0141 |
| 5 | PDS5A | Sororin-cohesin complex | 0.0327 |
| 6 | ZWILCH | ACTB-ANP32A-C1QBP-PSMA1-PTMA-PSMA1 complex | 0.0497 |
| 7 | NCAPG | Condensin I-PARP-1-XRCC1 complex | 0.0141 |
| 8 | ATP5F1 | F1F0-ATP synthase | 0.0065 |
| 9 | NDUFC2 | Respiratory chain complex I | 0.0427 |
| 10 | NCKAP1 | Wave-2 complex | 0.0284 |
| 11 | PSMD6 | PA700 complex | 0.0026 |
| 12 | MYBBP1A | Nop56p-associated pre-rRNA complex | 0.0141 |
| 13 | TIMM44 | HSPA9-GRPEL1-GRPEL2-TIMM44 complex | 0.0261 |
| 14 | HNRNPF | C complex spliceosome | 0.0427 |
| 15 | SF3B1 | 17S U2 snRNP，Spliceosome | 0.0331 |
| 16 | CCT7 | CCT complex | 0.0355 |
| 17 | CCT6A | CCT complex | 0.0427 |
| 18 | CCT5 | CCT complex | 0.0141 |
| 19 | CCT4 | CCT complex | 0.0142 |
| 20 | CCT3 | CCT complex | 0.0142 |

Proteins listed had (1) a calculated P value in 3 replicates; (2) were either stabilized or destabilized in 3 replicates; (3) had an intensity shift of ≥ 10%.
